# Educational Disparities in Preventable Deaths: Do They Explain the Longevity Gap Between Mexico and Spain?

**DOI:** 10.1177/08982643241303585

**Published:** 2024-11-26

**Authors:** Octavio Bramajo, Víctor M. García-Guerrero, Iñaki Permanyer

**Affiliations:** 1 Sealy Center on Aging, 441907 The University of Texas Medical Branch, Galveston, TX, USA; 2 560763 Centre d’Estudis Demogràfics, Universitat Autónoma de Barcelona, Bellaterra, Spain; 3 Center for Demographic, Urban and Environmental Studies, 27738 El Colegio de Mexico A.C., Mexico; 4 ICREA- Institució Catalana de Recerca i Estudis Avançats

**Keywords:** life expectancy, preventable mortality, educational attainment, Spain, health inequities

## Abstract

**Objective:**

Determine how preventable causes of death contribute to the life expectancy gap between Mexico and Spain.

**Methods:**

We used a linear integral decomposition to analyze the impact of preventable mortality on life expectancy between ages 30-75 (temporary life expectancy) between Mexico and Spain in 2018. Additionally, we computed cause-deleted life tables to estimate potential gains in temporary life expectancy. Analyses were stratified by educational attainment, sex, and age.

**Results:**

Low-educated Mexicans showed the largest gains in temporary life expectancy from removing preventable deaths (3.4 years for males, 1.6 for females), partially explaining the gap with Spain. Removing these deaths would close the gap almost entirely due to a higher relative decrease for middle- and high-educated individuals.

**Discussion:**

While access to adequate healthcare is crucial for improving population health, appropriate non-medical public policies can significantly reduce mortality disparities between Mexico and Spain, especially for individuals from higher educational backgrounds.

## Introduction

### On Preventable Mortality

The sustained increase in global life expectancy is arguably one of the most remarkable demographic events of the latter half of the 20^th^ century and beyond (Oeppen & Vaupel, 2002; Riley, 2015). This increase has made living longer a common expectation rather than an exception. The epidemiological transition theory emphasizes the shift over time from a higher proportion of deaths linked to infectious diseases, hunger, and violence to deaths related to chronic, long-term conditions (McKeown, 2009; Omran, 1971). However, some deaths remain preventable through comprehensive public policies, including timely medical interventions and political decisions. Broadly defined as “preventable deaths” by the OECD (European Commission, 2022), these include both external deaths, those resulting from other causes associated with the adoption of risky behaviors across the life course and those that could be averted with adequate treatment. Such deaths could be avoided through policies aimed at preventing these behaviors and mitigating their associated risks.

Similar to amenable mortality, preventable mortality focuses on premature deaths as conditions that could have been avoided with timely and effective healthcare (Gianino et al., 2017; Nolte & McKee, 2011; Rutstein et al., 1976, Mühlichen, 2019; Mühlichen et al., 2019). However, unlike amenable mortality, preventable mortality refers not only to deaths that can be avoided through timely care or treatment in a medical facility but also to deaths influenced by broader societal factors. Countries with high levels of violence or deaths from external causes, drug abuse, and varying political, institutional, and economic contexts experience higher levels of preventable mortality due to inadequate social policies that either do not prevent these deaths from occurring or could even actively contribute to their occurrence.

Furthermore, in addition to external deaths, preventable mortality also includes deaths from conditions that could be mitigated or prevented through public policies, such as lung cancer (through anti-smoking legislation or cessation of smoking) and diabetes (through policies promoting healthy eating habits and physical activity). Because these policies benefit the majority of the population, preventable causes of death are generally easier to address with effective public measures compared to other causes that may require more specialized and less cost-effective interventions. Therefore, countries with a high burden of preventable mortality can achieve substantial gains in life expectancy by addressing these preventable causes of death.

### Preventable Mortality in Mexico and Spain

Some countries in Latin America are among those most affected by preventable mortality due to violence and external causes. The largest of these Spanish-speaking countries in terms of population is Mexico, which is experiencing a singular epidemiological transition alongside an accelerated aging process (Frenk et al., 1991; Soto-Estrada et al., 2016). Characterized by high premature mortality primarily driven by external causes, Mexico has experienced stagnation in life expectancy over the past 20 years, largely due to violence resulting from the “war on drugs” that has affected its population since the turn of the 21^st^ century (Aburto et al., 2016; Canudas-Romo et al., 2014; Dávila-Cervantes & Pardo-Montaño, 2018). Alongside these deaths, Mexico also faces an increasing burden of non-communicable diseases, similar to other Latin American countries (Hambleton et al., 2023; Stuckler, 2008). Specifically, there has been an increase in mortality from endocrine-metabolic conditions (Rojas-Martínez et al., 2024). However, other preventable causes of death, such as lung cancer, have shown declines in mortality (Rizo-Ríos et al., 2015; Rojas-Martínez et al., 2019), indicating that this trend is uneven.

Other countries, such as Spain—currently a leader in longevity—also experienced high levels of preventable mortality before the 2000s due to causes such as drug-related fatalities, traffic accidents (Brugal et al., 2004; Fuente et al., 2006; González-Pérez et al., 2015), and certain neoplasms like lung cancer (Aragonés et al., 2009; Levi et al., 2007), partly attributable to the lack of anti-tobacco policies (Fernández et al., 2009). Before the 2000s, life expectancy in Spain grew more slowly than in Mexico, in part due to higher levels of preventable causes of death. However, since the turn of the century, Spain has significantly reduced its burden of preventable deaths (Cayuela, Asuero Llanes et al., 2020; Cayuela, Sánchez Gayango et al., 2020; González-Pérez et al., 2015; Nolasco et al., 2018). As a result, Spain’s standardized preventable mortality rate in 2015 was 100 per 100000 deaths, significantly lower than Mexico’s 215 per 100000 and the United States’ 181 per 100000, and 25% below the European Union average (Eurostat, 2017; Organisation for Economic Co-operation and Development, 2024).

Due to its high levels of life expectancy (United Nations Department of Economic and Social Affairs Population Division, 2022) and current low levels of preventable mortality, Spain can be considered a best-practice scenario within the Ibero-American world, which includes Spanish- and Portuguese-speaking countries. Despite this, until the onset of the Mexican war on drugs in 2006, both Spain and Mexico had similar growth rates in life expectancy: between 1990 and 2015, life expectancy at age 30 for both sexes increased by 2.5 years in each country (United Nations World Population Prospects, 2022). However, between 2005 and 2018, life expectancy at age 30 grew by 2.5 years in Spain but only 0.4 years in Mexico (United Nations World Population Prospects, 2022). This suggests that if Mexico were to reduce its burden of preventable deaths, it could significantly narrow the life expectancy gap with Spain and potentially return to a rate of increase comparable to or even surpassing that of Spain.

While the role of violence and external causes in the stagnation of life expectancy in Mexico has been examined, the impact of other preventable causes of death has not been fully explored. Specifically, if Mexico were to eliminate its burden of preventable mortality, including both violence and other preventable causes, would its life expectancy increase at a different rate, potentially closing the gap with a country like Spain? It remains unclear whether Spain’s current advantage over Mexico is due to its success in reducing preventable mortality or if other factors play a role. Furthermore, would Spain’s advantage diminish if the burden of preventable deaths were significantly reduced in Mexico and other Ibero-American countries?

The primary objective of this study was to quantify the burden of preventable causes of death in Mexico and assess whether these factors significantly contributed to the life expectancy gap with Spain. Additionally, the study aimed to determine the specific contributions of external causes and other preventable causes of death to contemporary mortality in Mexico.

### The Role of Age, Sex, and Socioeconomic Status in Preventable Mortality

Importantly, the burden of preventable causes of death does not affect all individuals equally. This burden, which includes both external and other preventable causes, is influenced by factors such as age, sex, and socioeconomic status (SES). Preventable deaths, like all-cause mortality, are more likely to be associated with lower social standing throughout the life course. SES has long been recognized as a crucial factor in analyzing disparities in health and mortality. According to the theory of fundamental causes (Link & Phelan, 1995; Phelan et al., 2010), SES is a key determinant of multiple and ever-changing mechanisms of health inequalities, including mortality. These mechanisms involve, but are not limited to, knowledge, exposure to harmful environments or activities, and a range of risks accumulated over the life course (Ferraro & Shippee, 2009), with SES serving as a fundamental cause.

Mexico has a highly stratified society, which has clear implications for health inequality. Previous studies have shown a clear gradient in mortality to the detriment of individuals with lower SES, mostly concentrated among younger age groups (Gómez-Ugarte & García-Guerrero, 2023; Muradás Troitiño, 2010; Sánchez et al., 2019). At more advanced ages, however, the SES gradient in mortality tends to diminish (Rosero-Bixby, 2018), suggesting that younger age groups experience the bulk of health inequality. Additionally, healthcare availability is a concern, as individuals without access to healthcare have higher mortality rates (Gómez-Ugarte & García-Guerrero, 2023). Therefore, this study also aimed to determine whether the burden of preventable mortality is distributed unevenly across educational levels and whether addressing this burden could significantly reduce disparities across educational groups.

### Previous Studies

Previous studies in other Latin American countries have also identified a social gradient in mortality based on educational attainment, disadvantaging adults with lower SES (Bramajo & Grushka, 2020; Sandoval et al., 2022; Sandoval & Turra, 2015). Although direct comparisons between Mexico and Spain are limited, a notable study (González-Pérez et al., 2015) examined mortality rates for specific causes classified as preventable by the Eurostat-OECD (see Appendix Table 1A for a comprehensive list), focusing on conditions such as diabetes, traffic injuries, ischemic heart diseases, and certain neoplasms (not all cancers are considered preventable under this classification). The findings revealed a stark contrast: between 2000 and 2010, Spain successfully reduced death rates for these causes, whereas in Mexico, mortality rates for most of these conditions either stagnated or increased. Interestingly, for some specific causes, such as certain cancers and traffic injuries among females, both countries exhibited similar levels and trends over time (González-Pérez et al., 2015).

Similar to Mexico, previous studies in Spain have found that individuals with a lower SES (using educational attainment as a proxy) also had a lower life expectancy (Bramajo et al., 2022; Permanyer et al., 2018; Trias-Llimós & Spijker, 2022). In Spain, mortality gradients across SES can be considered modest, with gaps in life expectancy between educational groups ranging between 2.5 and 3.5 years. These gaps are much smaller compared to those in Mexico (Gómez-Ugarte & García-Guerrero, 2023), where differences between individuals with the highest and lowest levels of education range from 6 to 7 years. The gaps in Spain are also smaller than those observed in the United States (Montez et al., 2019; Sasson, 2016) and in northern European countries (Van Raalte et al., 2011, 2018). Furthermore, Spain’s robust health system, supported by strong primary care (Bernal-Delgado et al., 2018), likely contributes to its recent low levels of preventable mortality.

While we acknowledge that preventable mortality is generally more prevalent among individuals with lower SES, it is unclear whether the contributions of different types of preventable mortality (external and other causes) vary by age. Violence and external causes typically affect younger males, but it is uncertain if these factors affect males with low education and middle education in the same way, although it is reasonable to assume that those with higher education are less affected. Furthermore, other preventable causes of death, such as most infectious diseases, some forms of cancer, and certain heart diseases, may exhibit less pronounced age-specific patterns across both sexes.

Therefore, in addition to establishing the overall burden of both types of preventable mortality, this study also examined related factors of health inequality, such as age and socioeconomic status, to better understand their roles in the life expectancy gap between Mexico and Spain.

## Data and Methods

### Data Sources

We used cross-sectional mortality data from Spain and Mexico for 2018, analyzing it by educational attainment as a proxy for SES.

For Mexico, we used five-year age group population exposures and death rates from the Demographic Conciliation data of the National Population Council (CONAPO by its acronym in Spanish). These life tables, which have been verified and adjusted using previous and posterior census data, vital statistics, and other registers, reflect the midpoint between the 2020 census and the 2015 postcensal survey, which was in 2018. Based on these population counts and age-specific death rates by sex, we established the proportion of deaths by cause (external, other preventable causes, and non-preventable causes) and by educational attainment using proportional estimates from vital statistics. Similar procedures have been used in previous studies to approximate the mortality proportions by educational attainment based on observed death rates (Beltrán-Sánchez & Andrade, 2013; Gómez-Ugarte & García-Guerrero, 2023). The share of unknown deaths by cause, categorized by educational attainment, was proportionally distributed across age groups. The age-specific death rates by sex and educational attainment for each country are detailed in Appendix Figure 1(A).

For mortality data and population exposures in Spain by educational attainment, we used aggregate mortality data and population counts from the Spanish National Institute of Statistics (Instituto Nacional de Estadística or INE), which have been used in previous studies (Bramajo et al., 2022; Permanyer et al., 2018; Trias-Llimós & Spijker, 2022). The INE employs a matching algorithm to link registered deaths with population databases, including censuses, municipal population registers, the Ministry of Education, and the Public State Employment Service, to obtain death counts by educational attainment when available. The INE also provides total population estimates by sex, age, and educational attainment upon request. Using these registers, we determined death counts and population exposures for the chosen period at the aggregate level. However, unlike the data for Mexico, only all-cause mortality by education is available for Spain.

Preventable deaths were defined according to the OECD/European Commission suggested classification (European Commission, 2022). A list of ICD-10 codes corresponding to the definitions of other preventable and external deaths is provided in Appendix Table 1A. Originally, the definition of preventable deaths was based on a modification of Rutstein et al.’s (1976) list of amenable causes of death, which focused on mortality that could be avoided with timely medical attention. However, since some preventable deaths can be avoided through precise non-medical policies, we adopted the broader definition of preventable deaths instead. While external deaths are included in this category, their impact was also considered separately due to the high levels of violence experienced in Mexico in recent years. The share of age-specific preventable and external deaths in both countries is shown in Appendix Figure 2(A).

After some testing, we selected three educational attainment categories: “low,” “middle,” and “high.” The “low” category represented completion of basic mandatory education (levels 0-2 in the UNESCO ISCED-11 classification, or less than 9 years of schooling), the “middle” category included high school education (levels 3-4 in ISCED-11, indicating between 9 and 12 years of schooling), and the “high” category referred to higher education attained after age 18 (levels 5-8 in the UNESCO ISCED-11 classification, or more than 12 years of schooling). Given the relatively recent educational expansion in both countries, we believed these categories clearly distinguished between individuals with basic versus post-basic education, reflecting differences in status and opportunities. The distribution of educational attainment across countries is presented in Appendix Table 2A, with both countries showing similar proportions in the middle education group, with fluctuations in the low and high education groups.

Given the availability of educational data, we used age 30 as the starting point for our estimations because mortality estimates below this age are not available for Spain. The large amount of deaths and population exposures in both countries would provide results that should be equally robust by starting with a different age group, such as age 25 or 35. That being said, studies on preventable mortality have typically considered deaths occurring before age 75, so we chose this age for the upper truncation in our analyses (European Commision, 2022; Mühlichen, 2019; Nolte & McKee, 2011).

### Measures

Since definitions of preventable mortality are restricted to deaths occurring before age 75 (OECD, 2024) and educational data is available starting at age 30, we computed temporary life expectancies (TLE30-75) between ages 30 and 75 to analyze differences in mortality (Preston et al., 2001), separately by sex and education across countries. Unlike life expectancy (that considers the lifespan that a hypothetical cohort would live until extinction), temporary life expectancy (also known as partial life expectancy) only estimates the average number of years lived between certain age groups (Arriaga, 1984). In a life table, TLE30-75 can be expressed as the quotient of the sum of person-years lived ( $T_{30-75}$ in life table notation) between the ages comprehended by the temporary period (in this case, 30 and 75), divided by the number of survivors at the initial age ( $l_{30}$ using life table notation), with 45 years of life (75 minus 30) as the highest possible value (see equation (1)). A non-temporary life expectancy would have the same denominator but would use the sum of person-years lived from age 30 (in this case) to the end of the life table as the numerator. We assumed that deaths were distributed evenly across ages, so the value of a_x_ for each five-year age group was fixed at 2.5.

(1) $$TLE30-75=\frac{T_{30-75}}{l_{30}}$$

To establish the age-specific contribution of each type of death (external, other preventable, and other causes) to differentials in TLE30-75 across countries, we used a widely recognized linear integral decomposition method known as the Horiuchi decomposition (Horiuchi et al., 2008). This method is available in the DemoDecomp package in R (Riffe, 2018).

To determine the burden of external and preventable deaths in overall mortality and their gains in longevity, we adapted a second counterfactual procedure known as the associated single-decrement life table (Preston et al., 2001) or cause-deleted life table (Beltrán-Sánchez et al., 2008). This instrument presents a scenario in which certain causes or groups of causes of death (in this case, external and other preventable causes) are excluded (denoted as cause “ i ” in equation (2)) from the sum of deaths (represented with “ x ” in equation (2)). Using the approach proposed by Chiang (1968), we established gains in TLE30-75 attributed to the removal of external and other preventable deaths, comparing the TLE30-75 obtained in these counterfactual distributions with the baseline scenario (the one that includes all causes of mortality), as indicated in equation (2).

(2) $${TLE}_{30-75\;\left(x-i\right)}=\frac{T_{30-75\;\left(x-i\right)}}{l_{30\left(x-i\right)}}$$

Because we do not have the educational distribution of causes of death in Spain, we established gains in longevity by sex, cause (external, other preventable, and non-preventable causes), and education for Mexico, and by sex and group of causes for Spain. Appendix Figure 3(A) presents the age-specific distribution of death rates by sex, type, and education in Mexico, showing that non-external preventable deaths (other) and non-preventable deaths present similar educational gradients.

## Results

Table 1 presents the estimations of TLE30-75 by education and sex for Spain and Mexico in 2018. In Spain, males and females with high education had the highest values in TLE30-75, with 37.8 years for males and 41.6 years for females, indicating a potential loss of life of 7.2 and 3.4 years, respectively, from the maximum threshold. In contrast, Mexico showed the lowest values in TLE30-75, with low-educated males having the lowest of all estimates at 32.7 years, representing a temporary loss of life of 12.3 years. Across countries, the largest TLE30-75 gap was observed in the low education group, with a gap of 4.8 years for males and 3.3 for females, which was nearly double or triple the gap found in the high education group.

**Table 1.** TLE30-75 in Spain in Mexico by Sex and Educational Attainment, 2018 (in Years).

Country	Males, low	Males, mid	Males, high	Males, all	Females, low	Females, mid	Females, high	Females, all
Mexico	32.7	36.2	37.8	34.3	37.4	39.6	40.4	38.1
Spain	37.5	39.0	40.3	38.7	40.7	41.2	41.6	41.3
Gap	4.8	2.8	2.5	4.4	3.3	1.6	1.2	3.2

Table 2 presents the cause-specific decomposition of the TLE30-75 differences between Spain and Mexico, which together accounted for the total gap between the countries. On average, external and other preventable causes of death explained almost 70% of the gap for males and slightly more than 40% for females. For males, the contributions of external and other preventable causes in the gap were evenly distributed, with each component representing one-third of mortality. For females, non-external preventable causes were responsible for almost the entire contribution of preventable causes in the TLE30-75 gap, with external deaths representing only 0.2 years of life.

**Table 2.** Cause-Specific Contribution in TLE30-75 Gaps Between Spain and Mexico by Sex and Educational Attainment.

Type	Males, low	Males, mid	Males, High	Males, all	Females, low	Females, mid	Females, high	Females, all
External	2.0	1.1	0.6	1.5	0.2	0.2	0.1	0.2
Other preventable	1.4	1.1	1.1	1.4	1.2	0.4	0.2	1.1
Non-preventable	1.4	0.6	0.8	1.4	1.9	1.0	0.9	1.9
Total gap (years)	4.8	2.8	2.5	4.4	3.3	1.6	1.2	3.2
% of gap explained by preventable deaths	70.8	78.6	68.0	65.9	42.4	37.5	25.0	40.6

In the analysis stratified by education (see Table 2), among males with low education, the impact of external causes was the largest of all contributions across groups: 2.0 years of the 4.8-year gap were explained by external causes. When combined with the 1.4 years attributed to the other preventable causes, this implied that more than three-quarters of the TLE30-75 gap with Spain could be explained by these causes of death. For the middle and high education groups, the burden of other preventable causes and non-preventable causes was similar. The key difference between these groups was that the contribution of external deaths was half a year higher for the middle education group. However, in all cases, the percentage of preventable deaths that explained the TLE30-75 gap with Spain exceeded 65%, with the middle education group presenting the largest share at 78.6%, arguably because of the contribution of external deaths and the relatively smaller contribution of non-preventable deaths.

Among females, the low education group had the largest share of the TLE30-75 gap between Mexico and Spain explained by external and other preventable causes, at 42.4%. In contrast, the high education group attributed only one-quarter of the total gap to these causes of death. It is worth noting that, unlike males, the burden of external deaths is quite small across all educational groups for females. However, for low-educated females, other preventable causes of death contributed more to the gap than for those in the middle and high education groups, with differences of 0.8 and 1 year higher, respectively.

**Figure 1.**
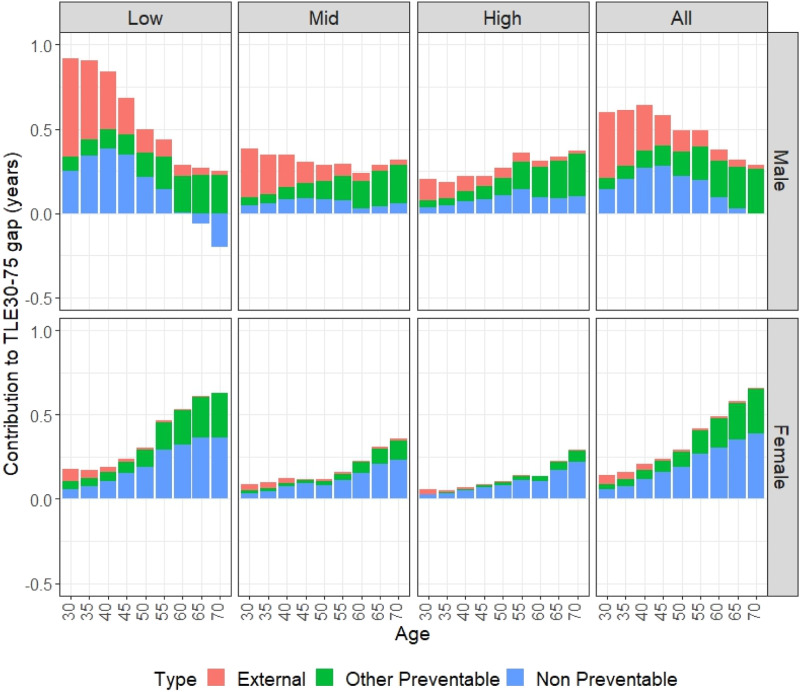
Age and cause-specific contribution in TLE gaps between Spain and Mexico by sex and educational attainment.

Figure 1 shows the age-specific contribution to the TLE30-75 gap across countries by cause and educational attainment. For males, external deaths presented a descending pattern with age, indicating that the largest potential losses of life occur at younger ages, particularly for those with low and middle education, with the majority of deaths occurring before age 50. The age-specific pattern for other preventable deaths was similar across all educational groups, with the largest gains observed after age 50. In contrast, the contribution of non-preventable deaths varied by educational attainment. In the low education group, the largest gains were found at younger ages, with a slight advantage for Mexico after age 65. For the middle and high education groups, the contributions of non-preventable deaths were more evenly distributed across all age groups.

The bottom half of Figure 1 suggests that the patterns of contribution for other preventable and non-preventable deaths were similar for females, with both showing an increasing contribution by age. This finding indicates that, unlike their male counterparts, the contribution of non-preventable causes for females was more pronounced at older ages. Additionally, gains attributed to external causes were minimal and primarily affected younger age groups, while the contribution from other causes increased with age, peaking after age 50. However, as shown in Table 2, the contribution of other preventable deaths seemed to be larger for the low education group and the average.

Table 3 presents the counterfactual gains in TLE30-75 by removing both external and other preventable causes of deaths in Mexico. We also performed the same counterfactual analysis for the average population of Spain by sex, without stratifying by education. In almost all cases, the gains from removing preventable mortality were larger than those from removing external causes. The exception was males with low education, for whom the gains in TLE30-75 were more substantial by removing external deaths: 2.3 years of life gained compared to 1.1 years from removing other preventable deaths. However, this was not enough to close the gap with their Spanish counterparts, with nearly one-third of the 4.8-year gap remaining. Due to the weight of the low education group in the average composition, the gains from removing external causes were also greater than the gains from removing other preventable causes in the average male population, reducing the gap with Spain to 0.9 years, representing a reduction of 80% from the initial 4.4-year gap. For the middle education group, the gains obtained by removing both types of preventable causes would be enough to entirely close the gap with their Spanish counterparts, while for the high education group, the gap would be reduced to less than half a year.

**Table 3.** Gains in Temporary Life Expectancy Between Ages 30 and 75 (TLE30-75) for Mexico and Spain by Removing External and Other Preventable Mortality, Separately by Sex and Educational Attainment (in Years).

Country	Sex	Education	Total life expectancy	Gains without external	Gains without preventable	Total gains	New gap with Spain
Mexico	Males	Low	32.7	2.3	1.1	3.4	−1.4
		Mid	36.2	1.4	1.5	2.9	0.1
		High	37.8	0.7	1.4	2.1	−0.4
		All	34.3	1.7	1.2	2.9	−0.9
	Females	Low	37.4	0.3	1.3	1.6	−1.7
		Mid	39.6	0.3	1.1	1.4	−0.2
		High	40.4	0.2	0.9	1.1	−0.1
		All	38.1	0.3	1.2	1.5	−1.7
Spain	Males	All	38.7	0.4	0.5	0.9	−0.4
	Females	All	41.3	0.1	0.7	0.8	−0.1

For females, removing external and other preventable causes of death would result in a TLE30-75 gain of 1.6 years, with slightly more than half of the gap with Spain remaining. However, like their male counterparts, removing both external and other preventable causes would almost eliminate the TLE30-75 gap for the middle and high education groups, with preventable deaths carrying most of the gains. For Spain, the burden of external causes of death was minimal, contributing less than half a year of life for males and 0.1 years for females. Additionally, preventable deaths accounted for less than a year of life. Removing both groups would have yielded average gains of 0.9 years for males and 0.8 years for females, which were 2.0 and 0.7 years less, respectively, than the gains observed for their Mexican counterparts.

## Discussion

### Summary of Findings

This study examined the burden of preventable mortality in contemporary Mexico and, to a lesser extent, Spain, focusing on both external deaths and other preventable causes. Additionally, this study stratified the increases in life expectancy by sex and educational attainment, exploring the age-specific patterns of both types of preventable mortality and determining the contribution of preventable deaths within each group.

Because living long and healthy lives is normatively desirable, reducing the share of preventable deaths would reflect societal progress in preventing deaths that are treatable or avoidable through healthcare, governmental, or other societal factors. In Mexico, life expectancy has stalled since the onset of the war on drugs. Although external causes of death, particularly among males with low education, are a significant factor, these deaths are largely concentrated under age 50. Beyond this age, other preventable deaths, such as those related to diabetes or various types of cancer, likely account for most of the temporary life expectancy gaps with Spain.

The results of this study show that preventable mortality (both external and other preventable causes) accounts for 65% and 40% of the observed gap in life expectancy between Mexico and Spain for males and females, respectively. Notably, in certain educational groups, these shares reach up to 78% for males and 42% for females. This finding indicates that for males, most of the observed gap is attributed to preventable deaths, whereas for females, more than half of the gap is explained by non-preventable mortality.

The age-specific patterns reveal that external deaths are predominantly concentrated under age 50, while other preventable and non-preventable causes of death are more prevalent after age 50, suggesting that targeting preventable causes of death, especially those occurring after age 50, could lead to substantial gains in life expectancy for Mexico. Furthermore, for males, both in absolute and relative terms, other preventable causes of death represented a higher burden of mortality than for females, reenforcing the idea that among females, non-preventable causes—potentially preventable with targeted policies or adequate healthcare—play a more significant role in the TLE30-75 gradient.

In estimating the gains in TLE30-75 for Mexico, removing both types of mortality would not fully close the gap for low-educated individuals but would nearly close it for those with middle and high education. Thus, while a hypothetical reduction in preventable mortality would result in a larger absolute gain in life expectancy for the low education group, the burden of non-preventable causes of death—also potentially avoidable with certain policies and focus—is also relevant in explaining the gap in life expectancy between Mexico and Spain. For middle and high education groups, the gradient attributable to non-preventable causes is less pronounced than for their low education counterparts, suggesting that targeted reductions in preventable causes would be more beneficial for these groups.

## Conclusions

The higher mortality rates in low education groups may be attributed to SES exacerbating health disparities in countries with high levels of social inequality, such as Mexico (Gómez-Ugarte & García-Guerrero, 2023; Sánchez et al., 2019). Furthermore, the lack of healthcare access is associated with worse health outcomes (Gómez-Ugarte & García-Guerrero, 2023), which may contribute to the higher share of preventable and non-preventable deaths observed in Mexico. Spanish females, by contrast, present smaller gradients in mortality by education (Bramajo et al., 2022; Permanyer et al., 2018), which may be related to the relatively low proportion of the population with unmet healthcare needs (Zueras & Rentería, 2020), which, in turn, is likely associated with the lower prevalence of preventable deaths (both external and non-external) observed in Spain.

The role of healthcare policies in reducing preventable deaths cannot be understated. The discontinuation of the Seguro Popular program in Mexico in 2019, designed to provide healthcare to individuals lacking social security benefits, is particularly noteworthy. The Seguro Popular was later replaced by other programs, currently the Health Services of the Instituto Mexicano del Seguro Social para el Bienestar. However, the effectiveness of these new programs has not yet been thoroughly assessed. This raises concerns that future health inequalities might worsen if these new programs prove less effective than the Seguro Popular in providing adequate healthcare.

While healthcare access issues also impact the health burden for Mexican males, they face an additional layer of preventable mortality due to a high share of external deaths. The recent surge in violence in Mexico has disproportionately affected males, contributing to stagnation and even reductions in life expectancy (Aburto et al., 2016; Canudas-Romo et al., 2017; Dávila-Cervantes & Pardo-Montaño, 2018). Eliminating both external and other preventable causes of deaths could lead to significant improvements in TLE30-75, potentially closing much of the gap with their Spanish counterparts. On one hand, external deaths are largely associated with violence, the drug trade, and inadequate safety policies that fail to adequately protect lives, reflecting broader non-health-specific issues rather than health-specific policies. On the other hand, the burden attributed to other preventable deaths is more closely linked to deficiencies in healthcare and sanitary policies. Additionally, individuals with higher education and SES may experience lower mortality rates due to greater health awareness and healthier behaviors, such as reduced smoking rates. While both external and non-external mortality are preventable, addressing them requires distinct and targeted public policies to effectively reduce the loss of life expectancy.

## Limitations and Final Comments

The limitations of this study primarily stem from defining preventable deaths using cross-sectional data from 2018, which lacks multiple observations for a larger comparison. A multistate framework based on available longitudinal data would provide clearer insights into health transitions and allow for a more accurate assessment of preventable deaths within the population. However, such data is rare and typically available only in high-income countries, which generally exhibit lower levels of preventable mortality. Other sources that consider health transitions, such as the Survey of Health, Aging, and Retirement in Europe and the Mexican Health and Aging Study, often do not record causes of death. Additionally, the definition of preventable mortality establishes a threshold age, which includes many individuals who are still alive, necessitating the use of Temporary Life Expectancy as an indicator for comparing mortality levels. Moreover, the lack of educational data for those under age 30 prevented us from analyzing external mortality in younger populations, which is significant in Mexico, thus limiting our analysis. At older ages, the burden of preventable deaths may also be slightly overestimated because physicians or administrative clerks might not capture other underlying conditions on death certificates (Désesquelles et al., 2010; Trias-Llimós & Permanyer, 2023). Finally, the study does not address how societal factors across countries might influence causes of death by education level, nor does it consider the potential role of genetic factors in mortality.

In addition, the educational gradient we estimated may vary across countries, as the implications of educational attainment for health can differ across countries. However, while changes in the share of deaths or our aggregate estimates are possible, the meaning of our findings should remain intact due to the robustness of life expectancy as an indicator for summarizing mortality at a population level.

This study underscores that reducing mortality extends far beyond healthcare; it demands comprehensive public policies that address a range of issues, from anti-smoking campaigns to curb lung cancer to political measures that reduce homicides. The failure of the military-driven approach since the start of the Mexican War on Drugs in 2006, which has led to tremendous loss of life, highlights the urgent need for alternative strategies. The high burden of preventable deaths is a major factor contributing to Mexico’s lower life expectancy compared to Spain, a world leader in longevity partly due to its low levels of preventable mortality. Furthermore, the disproportionate number of preventable deaths by educational attainment would also require certain specific strategies that focus on structural vulnerabilities, since non-preventable causes are also responsible for the Mexican lag for individuals with lower SES. Addressing preventable deaths through effective policy interventions is crucial for Mexico to achieve substantial gains in life expectancy in the coming years. Each preventable death that is not avoided represents a lost opportunity to close the gap and secure a healthier future for all Mexicans.

## Supplemental Material

Supplemental Material - Educational Disparities in Preventable Deaths: Do They Explain the Longevity Gap Between Mexico and Spain? Supplemental Material for Educational Disparities in Preventable Deaths: Do They Explain the Longevity Gap Between Mexico and Spain? by Octavio Bramajo, Víctor M. García-Guerrero and Iñaki Permanyer in Journal of Aging and Health

## Data Availability

Part of the dataset used in this study is not publicly available. However, it may be obtained from the corresponding author upon reasonable request.
