# Supplementary material for: Educational Disparities in Preventable Deaths: Do They Explain the Longevity Gap Between Mexico and Spain?
Source: J Aging Health. 2024 Nov 26;37(10):733–43. doi: 10.1177/08982643241303585 (PMC12541120; doi:10.1177/08982643241303585)
Supplement: Supplemental Material - Educational Disparities in Preventable Deaths: Do They Explain the Longevity Gap Between Mexico and Spain? [file sj-pdf-1-jah-10.1177_08982643241303585.pdf]

**Appendix:**

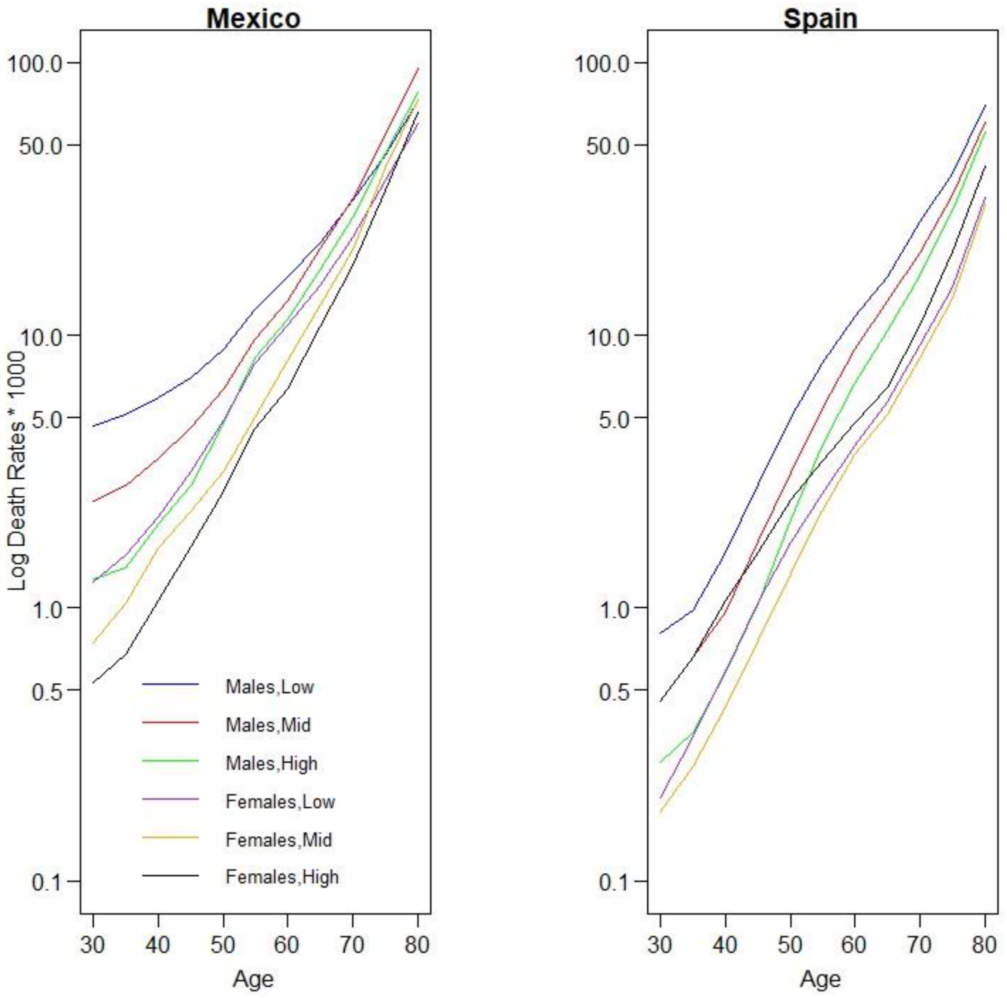


Figure 1A: Age-specific Death Rates by sex and educational attainment, Mexico and Spain.

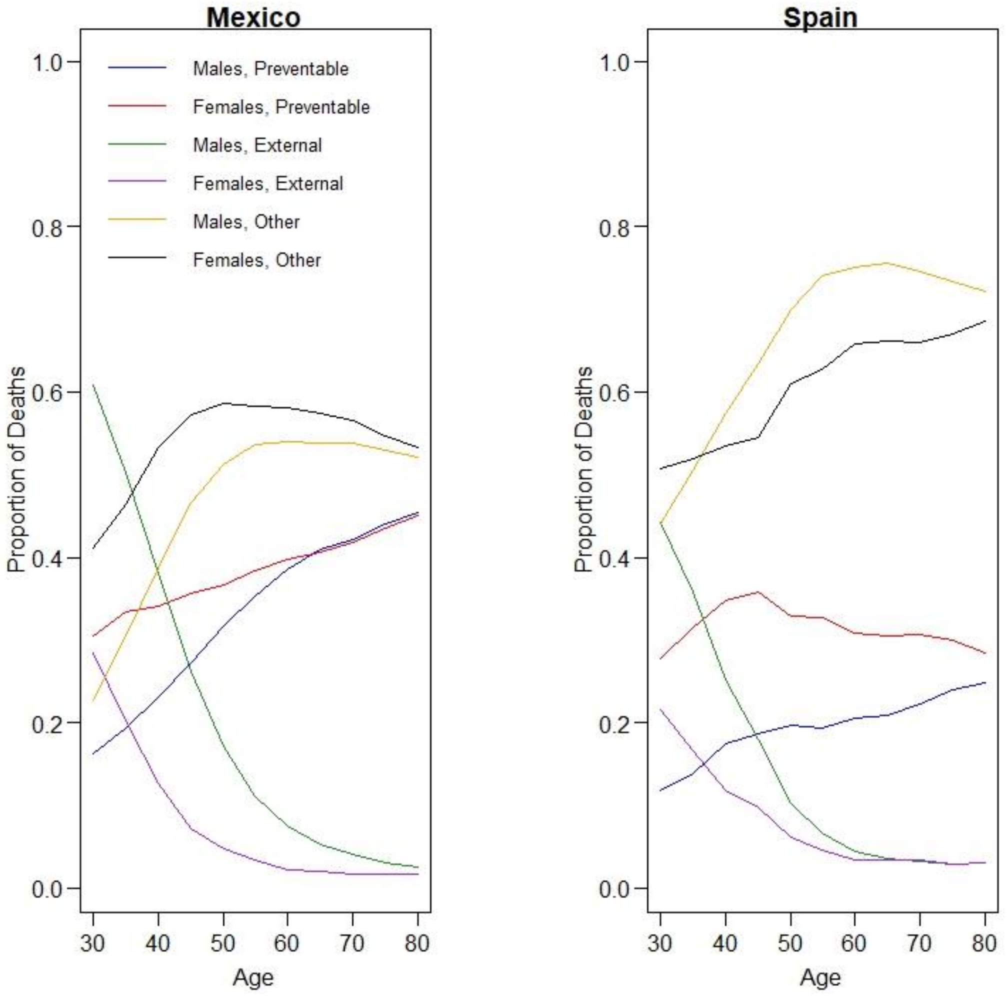


Figure 2A: Age-specific Proportion of Preventable, External and Rest of Deaths by sex , Mexico and Spain.

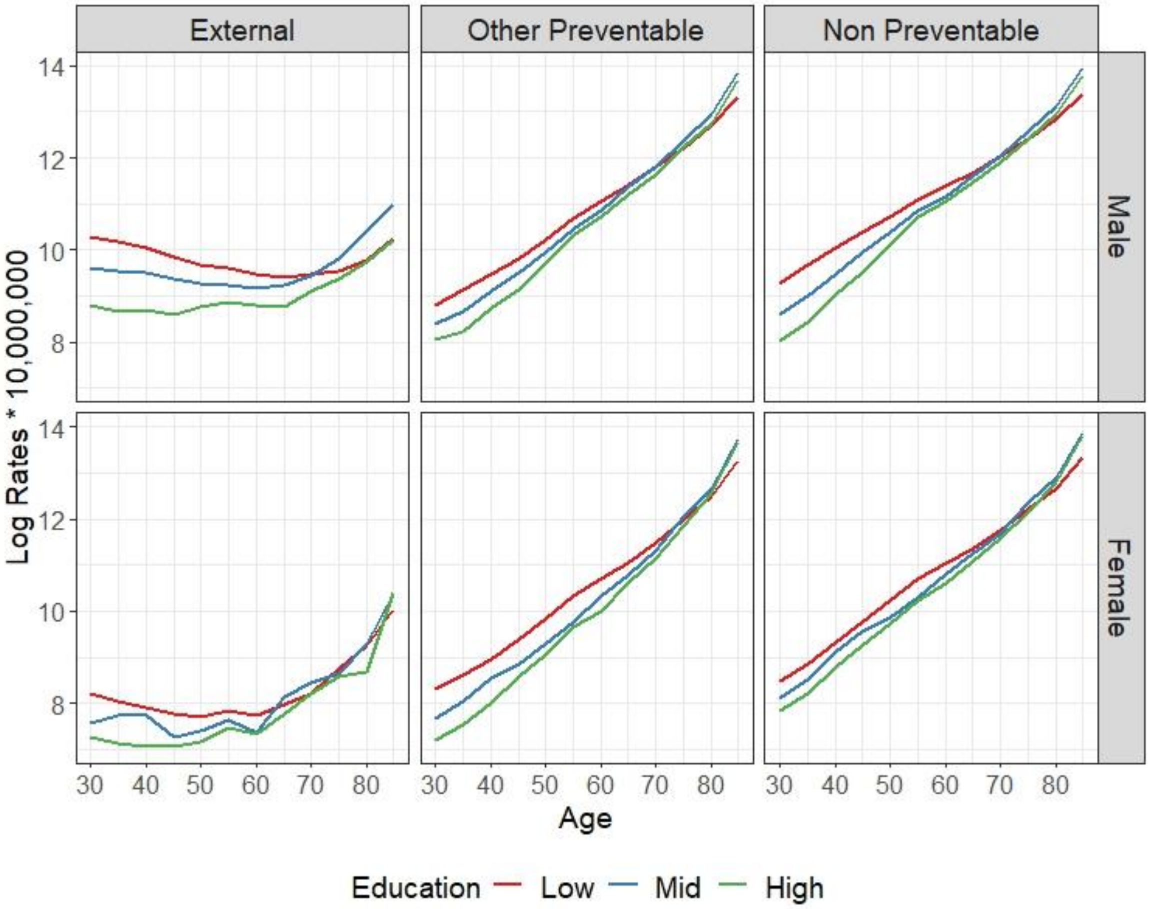


Figure 3A: Age-specific Death Rates of External, Other Preventable and Non-preventable deaths by sex and education, Mexico.

| Group | Cause of Death | ICD-10 Codes |
|---|---|---|
| Infectious and parasitic (Other Preventable) | Intestinal Diseases | A00-A09 |
| | Diphteria, Tetanus, Poliomyelitis | A35 A36 A80 |
| | Whooping Cough | A37 |
| | Meningococcal Infection | A39 |
| | Sepsis due to streptococcus pneumonia and sepsis due to hemophilus influenzae | A40.3 A41.3 |
| | Haemophilus influenza infections | A 49.2 |
| | Sexually transmited infections (except HIV/AIDS) | A50-60 A63-64 |
| | Varicella | B 01 |
| | Measles | B05 |
| | Rubella | B06 |
| | Viral Hepatitis | B15-19 |
| | HIV/AIDS | B20-24 |
| | Malaria | B50-54 |
| | Haemophilus and pneumococcal meningitis | G00 G00.1 |
| | Tuberculosis (50%) | A15-19,B90,J65 |
| Cancer (Other Preventable) | Lip, Oral cavity and pharynx cancer | C00-14 |
| | Oesophagaeal cancer | C15 |
| | Stomach cancer | C16 |
| | Liver Cancer | C22 |
| | Lung Cancer | C33-34 |
| | Skin cancer | C43 |
| | Mesothelioma | C45 |
| | Bladder Cancer | C67 |
| | Cervical cancer (50%) | C53 |
| Endocrine and metabolic (Other Preventable) | Nutritional deficiency anaemia | D50-53 |
| | Diabetes Mellitus (50%) | E10-14 |
| Circulatory (Other Preventable) | Aortic Aneurysm (50%) | I71 |
| | Hypertensive diseases (50%) | I10-13, I15 |

| | | |
|---|---|---|
| | Ischaemic heart disesaes (50%) | I20-25 |
| | Cerebrovascular diseases (50%) | I60-69 |
| | Other atheroschlerosis (50%) | I70-73.9 |
| Respiratory (Other Preventable) | Influenza | J09-11 |
| | Pneumonia due to Streptococcus pneumonia or Haemophilus influenza | J13-14 |
| | Chronic lower respiratory diseases | J40-44 |
| | Lung diseases due to external agents | J60-64,J66-70,J82,J92 |
| Pregnancy, childbirth and perinatal period and congenital malformations (Other Preventable) | Tetanus neonatorum | A33 |
| | Obstetrical tetanus | A34 |
| | Certain congenital malformations (neural tube defects) | Q00,Q01,Q05 |
| External (External) | Injuries | V01-99, W00-X39, X45-X59, X66-X84, Y16-Y34, X86-Y09 |
| Alcohol related and drug-related deaths (External) | Alcohol related and drug-related deaths | E24.4, F10,G.31.2, G62.1, G72.1, I42.6, K29.2, K70, K85.2, K86.0, Q86.0. R78.0, X45,X65, Y15,K73,K74.0-K74.2, K74.6,F11-F16,F18-F19,X40-X44,X85,Y10-Y14,X60 |

Table 1A: List of ICD-10 Codes Associated with other preventable and external mortality based on Eurostat-OECD classification.

| Country | Males, Low | Males, Mid | Males,High | Females, Low | Females, Mid | Females, High |
|---|---|---|---|---|---|---|
| Mexico | 59.7 | 18.0 | 22.2 | 62.5 | 17.0 | 20.3 |
| Spain | 50.1 | 19.8 | 29.9 | 50.7 | 19.0 | 30.2 |

Table 2A: Proportion of Educational attainment by sex, Mexico and Spain 2018.
